# A Protection Motivation Theory-Based Scale for Tobacco Research among Chinese Youth

**DOI:** 10.4172/2155-6105.1000154

**Published:** 2013-07-08

**Authors:** Karen MacDonell, Xinguang Chen, Yaqiong Yan, Fang Li, Jie Gong, Huiling Sun, Xiaoming Li, Bonita Stanton

**Affiliations:** 1 Department of Pediatrics, Wayne State University, Detroit, MI, USA; 2 Wuhan Centers for Disease Prevention and Control, Wuhan, China

**Keywords:** Protection Motivation Theory (PMT), Adolescents, Cigarette smoking

## Abstract

Rates of tobacco use among adolescents in China and other lower and middle-income countries remain high despite notable prevention and intervention programs. One reason for this may be the lack of theory-based research in tobacco use prevention in these countries. In the current study, a culturally appropriate 21-item measurement scale for cigarette smoking was developed based on the core constructs of Protection Motivation Theory (PMT). The scale was assessed among a sample of 553 Chinese vocational high school students. Results from correlational and measurement modeling analysis indicated adequate measurement reliability for the proposed PMT scale structure. The two PMT Pathways and the seven PMT constructs were significantly correlated with adolescent intention to smoke and actual smoking behavior. This study is the first to evaluate a PMT scale for cigarette smoking among Chinese adolescents. The scale provides a potential tool for assessing social cognitive processes underlying tobacco use. This is essential for understanding smoking behavior among Chinese youth and to support more effective tobacco use prevention efforts. Additional studies are needed to assess its utility for use with Chinese youth in other settings.

## Introduction

This study details the development and evaluation of a measurement scale for cigarette smoking behavior among adolescents in China based on the constructs of Protection Motivation Theory (PMT) [1]. The US-developed PMT may offer a promising theoretical framework for understanding tobacco use behavior in China. The central tenet of the theory is that people protect themselves based on their perceptions of four factors: the severity of a threatening event, the probability of the occurrence of threat, the efficacy of the recommended preventive behavior, and self-efficacy to adapt the recommended behavior [1]. Thus, PMT includes both threat and coping appraisals, making it particularly useful to explain why people engage in unhealthy behaviors, such as smoking, despite the well-known health risks.

**Figure 1:**
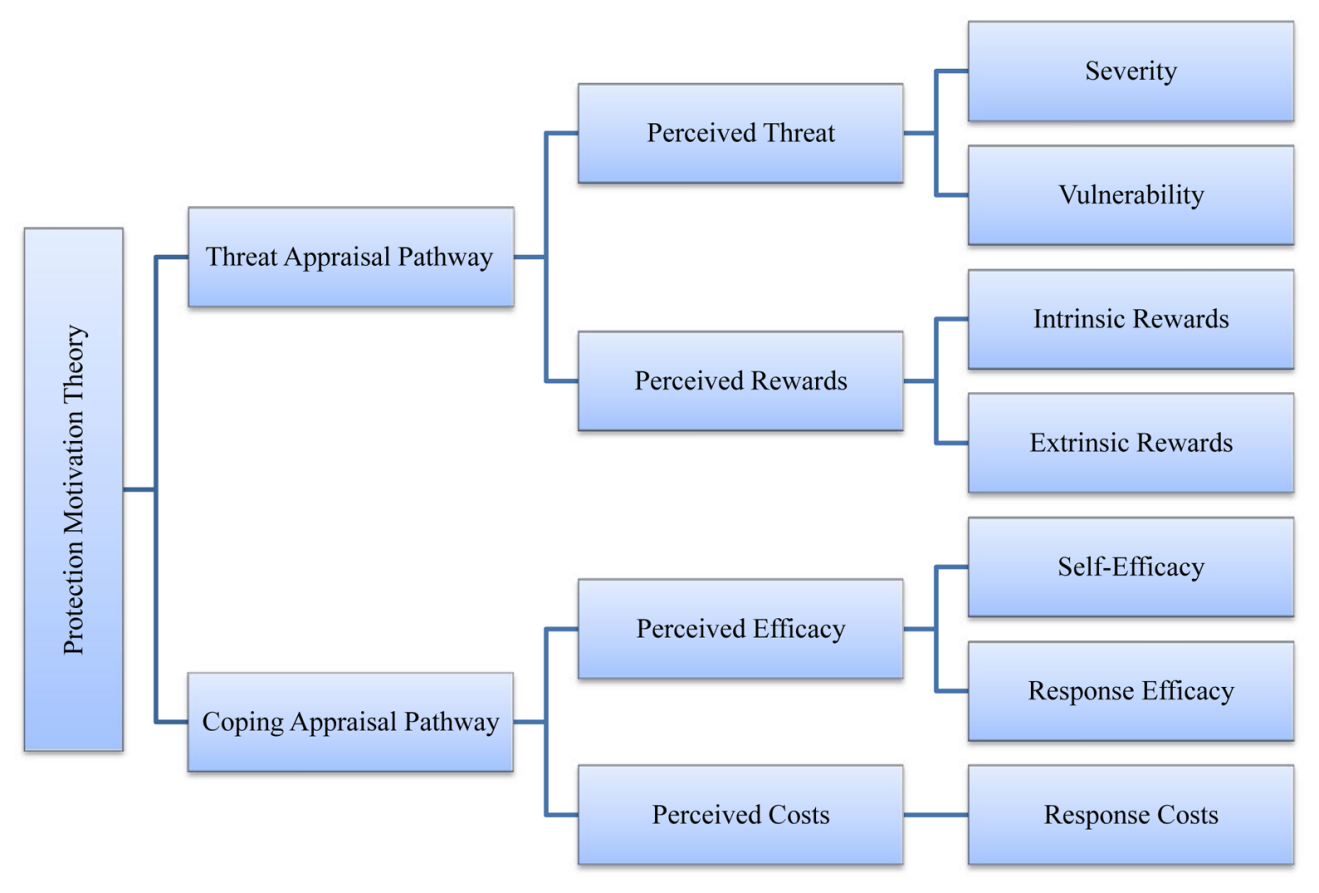
Overview of Central Constructs of Protection Motivation Theory (PMT). behavior. Increases in Perceived Efficacy and declines in Perceived Costs will decrease the likelihood of selecting a maladaptive risk behavior.

PMT was first introduced by Rogers to understand the effects of fear appeals on health-related attitude and behavior [1]. In the original PMT model, the influence of fear appeals was posited to be an important initiating factor influencing behavioral selection by an individual. The theory has undergone several revisions since it was first published [2–7]. In the current form, as shown in Figure 1, PMT consists of two highly correlated pathways: 1) Threat Appraisal, assessing the maladaptive behaviors (e.g. behaviors that lead an individual toward a health risk behaviors and/or to noxious consequences) and, 2) Coping Appraisal, assessing the ability to manage and avoid the threatened danger described by Threat Appraisal. The Threat Appraisal Pathway consists of four constructs in two groups, Perceived Threat and Perceived Rewards. Perceived Threat consists of two constructs, Severity and Vulnerability. Severity assesses the perceived negative consequences from a risk behavior, and Vulnerability assesses the perceived likelihood of the individual being affected by potential negative consequences. Perceived Rewards also includes two constructs, Intrinsic Rewards and Extrinsic Rewards. Intrinsic Rewards assesses the perceived positive physical and psychological effect from engaging in a risk behavior, and Extrinsic Rewards assesses the perceived positive social reactions or consequences of engaging in the risk behavior. Overall, perception of a greater threat will decrease the probability of selecting and engaging in a maladaptive behavior, whereas perception of a greater reward will increase the probability of selecting a maladaptive behavior.

The PMT Coping Appraisal Pathway consists of three constructs in two groups Perceived Efficacy and Perceived Costs. Perceived Efficacy consists of two constructs, Self-Efficacy and Response Efficacy. Self-Efficacy assesses the perceived ability to adapt a protective behavior, while Response Efficacy assesses the effectiveness of the protective behavior in lessening the health threat. Perceived Costs consists of one construct, Response Costs, which measures the perceived social, monetary, personal, time and effort costs from adapting the protective

### Research guided by PMT

PMT has been widely used in the West as a framework for researchers to investigate and understand a range of health-related behaviors [7,8]. As a theoretical guide, PMT has been used in etiological studies to investigate various risk and protective behaviors, including tobacco use [2,9], alcohol consumption [10,11], physical activity [12–14], self-care [15], safe and protective behaviors at the worksite [3,16], parental protective behavior [17], safe computing practices [18,19], and environmental hazard reduction [20]. As a conceptual framework, PMT has been utilized in intervention research in the West to develop and evaluate programs for purposeful behavior change, including interventions promote adherence to medical treatment regimens [21,22], prevent substance use [10,11], and discourage HIV risk behaviors and encourage HIV protective behaviors [23–26].

### Cigarette smoking in China: Prevalence and existing research

The current study is critical because cigarette smoking rates remain high among Chinese adolescents despite marked and ongoing tobacco control efforts [27,28]. Among Chinese adolescents, it is estimated that 33.3–47.8% of males and 12.8–24.3% of females have initiated smoking (i.e., have smoked at least once), and 15.0–18.30% of males and 1.7–4.0% of females are current smokers [29–33]. Despite substantial and documented tobacco control efforts in China, researchers have found only limited success in preventing or reducing tobacco use among adolescents [28,34]. One possible explanation for the lack of significant program effects could be that most tobacco prevention research and programs in China are not grounded in or guided by theory. Health behavior research conducted in more developed countries has consistently demonstrated the importance of a theory-based approach in etiological research to address health risk behaviors.

A theory-based approach is also critical for successful intervention research promoting purposeful behavior change and risk reduction [35–38]. We conducted a review and found only a few studies conducted in China (published in Chinese or English) that have been guided by a specific behavioral theory [27,31,39–46]. In addition, we found only one etiological study to date among those conducted in China that used the Theory of Reasoned Action and Planned Behavior as the guiding theoretical framework [47–49]. Among these studies, we identified only two intervention studies that included guiding theories related to social influences and media receptivity [27,50].

Despite the significance of many behavioral theories, including PMT, in explaining adolescent health behavior in general [7,24,25,51], and tobacco use behavior in particular [2,51], health behavioral research in China has largely been conducted without a guiding theoretical framework [52]. It is critical that health research be guided by theory, but there are barriers that must be addressed. One obvious challenge is the lack of relevant measurement scales of the essential constructs in the theoretical approach. In the present study, we developed and tested a PMT scale specifically for tobacco research in China. Here, we report our research findings on: (1) development of the culturally appropriate PMT scale for tobacco research with individual measurement items and subscales representing PMT constructs; (2) psychometric evaluation of the developed scale; and (3) assessment of the association of the PMT constructs with intention to smoke and actual smoking behavior. We see this as a critical first-step towards effective tobacco-control intervention in China; moreover, results suggest the potential for PMT-grounded research in other developing countries to promote cross-cultural tobacco research.

## Materials and Methods

### Development of the measurements for PMT constructs

The PMT scale developed for this study consisted of 21 items with three items for each of the seven PMT constructs. A seven-point Likert scale with 1 = “definitely disagree” to 7 = “definitely agree” was used for item scoring. The three items comprising *Severity* assess the perceived negative health consequences from smoking, e.g., “Smokers die earlier than nonsmokers.” The three items of the Vulnerability construct measure the perceived likelihood of being affected by tobacco-related negative health consequences, for example “I would get sick if I smoke.” The Intrinsic Rewards items focus on the perceived positive biological and psychological effects from smoking, for example “Smoking makes people feel comfortable.” Next, the three items compromising Extrinsic Rewards evaluate the perceived psycho-social benefits from smoking, including “Smoking is good for social networking.” The Self-Efficacy items assess an individual’s belief of his or her ability to refuse to smoke tobacco, for example “No one could persuade me if I do not want to smoke.” The Response Efficacy items are focused on an individual’s belief that nonsmoking is an effective approach for good health. Atypical item for this construct is “People will feel good by not smoking.” Finally, the three items compromising Response Costs evaluate the perceived psycho-social costs incurred from not smoking, for example “Refusing a cigarette offer is very impolite.”

Items were developed based on constructs of the PMT and from empirical data from related studies on adolescent health risk behaviors [24,25,29,53–58]. Following PMT theory and the published data, the lead author of this article drafted individual items (4–5 items per sub-construct). The drafted items were circulated among the co-authors and their colleagues for feedback to produce a draft version. The draft version was then reviewed by several middle school teachers in China for feedback and further revision to produce a pilot version. The pilot version was then tested among a small group of 10 Chinese middle school students to assess the readability and need for further revision.

### Research participants and procedures

Data were collected from a student sample in 2010. Participants were first-and second-year vocational high school students in Wuhan, China. Wuhan is located in central China and has a level of socioeconomic development close to the national average [59]. Students in year three were excluded because of their time commitments to graduation exams and post-graduation employment. We chose to sample vocational high school students because relative to students in regular high schools, health risk behaviors, including tobacco use, are significantly more prevalent among vocational high school students [60,61]. The three-year vocational high school education is established for middle school graduates who did not advance to regular high school. Chinese vocational high schools are exclusively run by the government following the same guidance and requirements established by the Chinese Ministry of Education for school size, student enrollment, curriculum, textbook, finance and administration. The purpose of the vocational high school program is to prepare students for a job by providing them with specific technical skills.

Students were sampled from a typical school with a medium school size and multi-occupational directions. Among the total 35 year-one and year-2 classes, 17 were randomly selected using the random digits method. All students in the sampled classes were invited to participate. Among the total 556 students in the sampled classes, three refused to participate, yielding a final sample of 553 (99.5%). Data were collected in the classroom using the Chinese Student Health Behavior Questionnaire (CSHBQ). The survey was administered by trained data collectors from Wuhan Centers for Disease Prevention and Control. It took approximately 20 to 30 minutes for most students to complete the survey. The research protocol was approved by the Human Investigation Committee at Wayne State University, United State and the Institutional Review Board at Wuhan Center for Diseases Prevention and Control, China. Approval of the school administration was obtained before students were sampled; in addition, informed consent was obtained from the students and their parents before the survey was administered.

### Scoring of the PMT constructs

Mean scores for the seven PMT constructs were computed respectively such that a higher score indicated a stronger belief or a greater perception. Scores for Perceived Threat were computed as the mean of Severity and Vulnerability. Scores for Perceived Rewards were the mean of Intrinsic Rewards and Extrinsic Rewards, and scores for Perceived Efficacy were computed as the mean of Self-Efficacy and Response Efficacy. Scores for Perceived Costs were equal the mean scores of Response Costs. Overall, scores for the Threat Appraisal Pathway were calculated as the difference between Perceived Threat and Perceived Rewards such that a higher score indicated a net surplus of perceived threat as compared to the rewards. Mean scores for the Coping Appraisal Pathway were computed as the difference between Perceived Efficacy and Perceived Costs such that a higher score indicated a net surplus of coping after the response costs were deducted.

### Measurement of cigarette smoking

Two smoking measures were used as outcome variables: intention to smoke and number of cigarettes smoked per day. The variable intention to smoke was assessed using data from the survey question: “How likely is it that you will smoke cigarettes in a year?” (1= very unlike and 5=very likely). The number of cigarettes smoked per day was measured using data from the question: “During the past 30 days, on the days you smoked, how many cigarettes did you usually smoke per day?” (# of cigarettes per day) To improve data distribution, the reported number of cigarettes per day was categorized into four levels with 1 = “0 cigarettes”, 2 = “1 cigarette”, 3 = “2 to 5 cigarettes”, and 4 = “more than 5 cigarettes”, following the method used in the Youth Risk Behavior Survey [62]. To validate reported smoking data, levels of carbon monoxide (CO) in the exhaled air were assessed using PiCO^+^™ (Smokerlyzer carbon monoxide monitor, USA). Our analysis indicated a significant correlation between the measured CO and the reported number of cigarettes smoked (*r* = 0.50, *p*<0.01). CO in the exhaled air reflects tobacco exposure in past 6–9 hours, and has been used as an effective biomarker for verification of self-reported smoking data [63,64].

### Data processing and analysis

**Figure 2:**
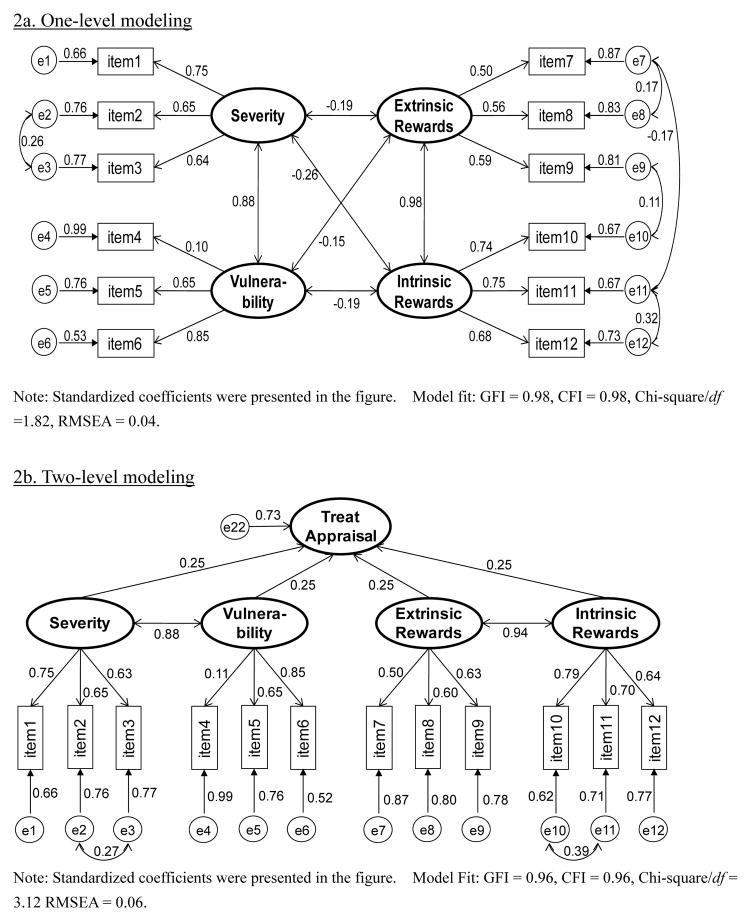
Measurement modeling analysis of the threat appraisal. Intrinsic Rewards, 0.61 for Extrinsic Rewards, 0.73 for Self-Efficacy, 0.68 for Responsive Efficacy, and 0.59 for Response Cost respectively. The α coefficient for Vulnerability could be increased from 0.48 to 0.70 if item 4 (perceived likelihood to be addicted to smoking) was deleted.

**Figure 3:**
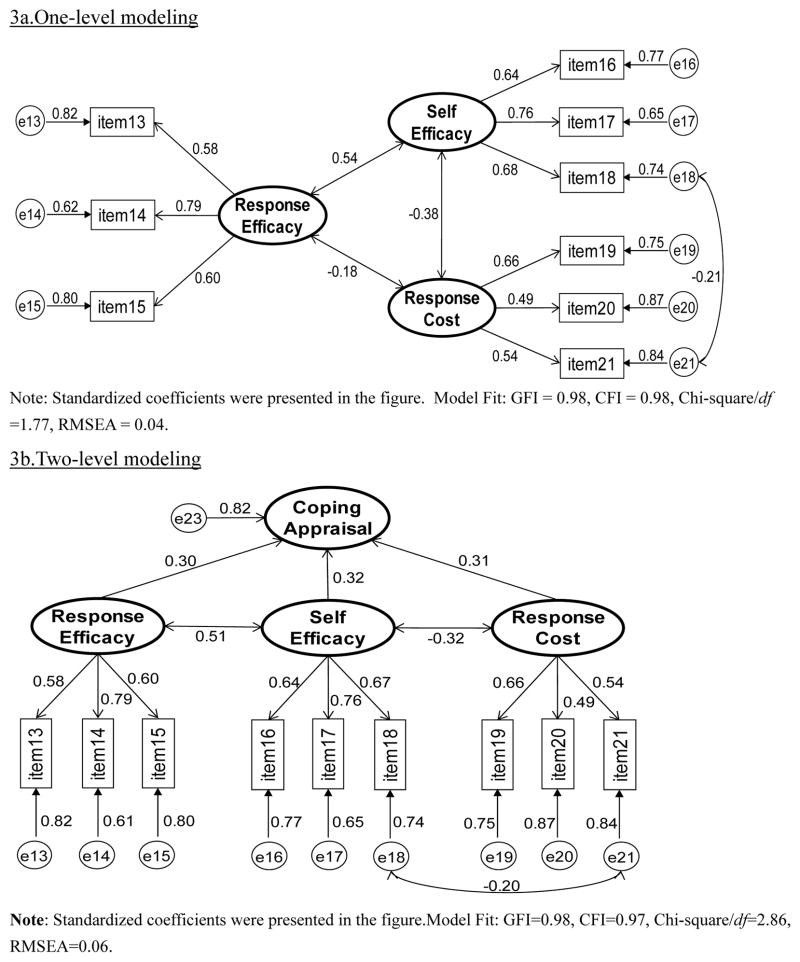
Measurement modeling analysis of the coping appraisal.

Survey data were manually entered using a 100% double-entry protocol to minimize data entry errors. Descriptive statistics such as rate, mean, and standard deviation, were used to summarize sample characteristics and students’ responses to the adapted PMT scale. Cronbach α and item-total correlation were used to assess the reliability of the individual PMT constructs. Measurement modeling via confirmative factor analysis was used evaluate the internal structure of the developed PMT scale. In the modeling analysis, the Threat Appraisal and the Coping Appraisal Pathways were analyzed separately (Figure 2 and 3). Four indices were used for assessing data-model fit: chi-square/df ratio (<2.0) [65], the root mean square error of approximation (RMSEA, ≤ 0.08 acceptable, ≤ 0.05 excellent) [66], goodness-of-fit index (GFI, ≥ 0.90 acceptable, ≥ 0.95 excellent), and comparative fit index (CFI, ≥ 0.90 acceptable, ≥ 0.95 excellent) [67]. Data processing and statistical analyses were completed using the software SAS version 9.13 (SAS Institute, Cary, NC).

## Results

### Sample characteristics

Sample characteristics and smoking behavior are summarized in (Table 1). Among the total 553 students, 275 (49.73%) were male, 300 (54.25%) were year-one students, and 253 (45.75%) were year-two students with a mean age of 16.31 years (SD = 1.12). More than 80% of the parents were married, 76.31% of the fathers and 67.27% of the mothers had a middle school or higher education. Twenty-two percent of the students reported either likely or very likely to smoke; 74.52% reported having not smoked any cigarette, and 13.5% smoked 5 or more cigarettes per day in the past month. Chi-square test indicated that on average boys had greater intention to smoke and smoked significantly more cigarettes than girls did (*p*<0.01 for both).

### Item responses and reliability of PMT constructs

Mean scores (SD), item-total correlations and Cronbach α of the PMT constructs are summarized in (Table 2). The number of students with missing data for individual PMT items varied from 2–5 (<1.00%). The mean score of the 21 items was 4.20 (SD=0.66), slightly greater than the middle point (=4.00) of the seven-point Likert Scale. The mean scores (SD) for the individual 21 items varied from 2.02 (1.66) for Item 19 (“A person may be isolated if he or she does not smoke) to 6.25 (1.48) for Item 1 (“The earlier a person starts smoking, the greater the harm”). The mean scores (SD) for the seven PMT constructs varied from 2.36 (1.44) for Intrinsic Rewards to 5.88 (1.36) for Severity. Cronbach α coefficients were 0.76 for Severity, 0.48 for Vulnerability, 0.80 for

### Measurement modeling analysis of PMT constructs

#### Threat appraisal

Results in Figure 2a indicate a satisfactory fit of the data with the one-level four-construct model for the Threat Appraisal Pathway (chi-square/df = 1.82, RMSEA = 0.04, GFI = 0.98, CFI = 0.98). Within the Threat Appraisal Pathway, Intrinsic Rewards and Extrinsic Rewards were highly correlated (covariate coefficient = 0.98); and so were Severity and Vulnerability (covariate coefficient = 0.88, *p*<0.01). Figure 2b depicts the two-level measurement model. Although slightly weaker than the one-level model with regard to the data-model fit indices, the two-level model fit the data well (chi-square/df = 3.12, RMSEA = 0.06, GFI = 0.96, CFI = 0.96).

#### Coping appraisal

Likewise, results in Figure 3a indicate a satisfactory fit of the data to the one-level Coping Appraisal Model (Chi-square/df = 1.77, RMSEA = 0.04, GFI = 0.98, CFI = 0.98). The two efficacy constructs were positively associated with each other while Response Costs was negatively associated with the two efficacy constructs. Results in Figure 3b also showed a satisfactory fit of the data to Two-Level Coping Appraisal Model (Chi-square/df = 2.86, RMSEA = 0.06, GFI = 0.98, CFI = 0.97).

### Correlation of PMT with cigarette smoking behavior

Results (Table 3) indicate that the seven PMT constructs individually and in groups, as well as the Threat Appraisal Pathway and the Coping Appraisal Pathway were each significantly correlated with intentions to smoke and the number of cigarettes smoked per day for the total sample and the subsamples of boys and girls, with a few exceptions. Perceived Threat and its two constructs were negatively associated with the intention to and the actual smoking behavior; Perceived Rewards and its two constructs were positively associated with the intention to and the actual smoking behavior; while higher Self-Efficacy and higher Response Efficacy were negatively associated with smoking intention and smoking measures and higher Response Cost was positively associated with smoking intention and smoking behavior measures.

## Discussion

In the current study, we developed and assessed a measurement scale for adolescent tobacco research in China based on Protection Motivation Theory [1–4,6]. The measurement scale was created by our research team with extensive feedback from researchers, school teachers and students. Results from our instrument evaluation study indicate that the scale reflects the inherent essential structure of PMT. It demonstrated acceptable reliability and, importantly, was significantly associated with intention to smoke and actual smoking behavior. The scale may be a critical tool for PMT-based tobacco research in China and an addition to the tools available for any theory-based intervention research in China [68].

The acceptability, reliability, and validity of the established PMT scale indicate the utility of PMT as a construct for measuring and understanding perceptions of smoking and related health consequences among Chinese adolescents. A number of studies among Chinese adolescents have reported data on perceived social rewards and negative consequences from smoking [31] and social influences on smoking initiation and progression [29,30,39,69–73]. Results of the present study add to this body of literature focused on social cognitive processes underlying smoking behavior. In this study, the central constructs of PMT significantly predicted smoking intent and behavior. The two primary PMT pathways, Threat Appraisal and Coping Appraisal, were associated with intentions to smoke and the number of cigarettes smoked per day. Consistent with PMT, youth who reported perceiving higher threat from smoking were less likely to report that they intended to smoke, and had less smoking behavior. In contrast, youth who perceived greater reward from smoking were more likely to report they intended to smoke and to actually smoke. In addition to etiological studies, the PMT scale can be used to measure potential mediating-and/or outcome variables when developing and evaluating intervention programs for tobacco use prevention among Chinese adolescents.

Despite the potential utility of the scale, there are notable weaknesses in the instrument. First, several items should be further refined to improve instrument responsiveness. For example, although the overall mean score of the 21 items = 4.20, close to the middle point 4 of the 7-point Likert scale (indicating good scale responsiveness); the mean scores for several items were either too small (e.g. item19 and item 12, <2.0) or too large (e.g. item1, item6, item14 and item15, >6.0). In addition, two constructs--Vulnerability and Response Cost-had low reliability. This should be addressed and tested in another sample to allow for further instrument development.

Despite these limitations, this study was the first to evaluate a scale measuring PMT constructs to understand cigarette smoking among Chinese adolescents. The scale provides a tool for assessing social cognitive processes underlying tobacco use. This is essential for understanding smoking behavior among Chinese youth to support more effective tobacco use prevention. Results suggest the utility of PMT theory and the PMT scale for understanding smoking behavior in a population at great risk for tobacco use, adolescents in China. Future research should further test the scale in other samples in China towards better understanding of PMT in a Chinese sample and additional scale refinement. The scale should also be tested and evaluated in other non-western countries towards the broader goal of promoting global efforts for tobacco control using theory-based measurement tools and intervention designs.

## Figures and Tables

**Table 1:** Characteristics of the study sample.

Characteristics	Boys		Girls		Total	
	N	%	N	%	N	%
Total	275	49.73	278	50.27	553	100.00
**Chronological age** (in years)						
15-	65	23.64	78	28.06	143	25.86
16-	87	31.64	90	32.37	177	32.01
17-	85	30.91	81	29.14	166	30.02
18-	38	13.82	29	10.43	67	12.12
Mean(SD)	16.37 (1.08)		16.24 (1.15)		16.31 (1.12)	
**School Grade**						
Year One	146	53.09	154	55.4	300	54.25
Year Two	129	46.91	124	44.6	253	45.75
**Parents’ marital status**						
Married	233	84.73	225	80.94	458	82.82
Divorce or others	42	15.27	53	19.06	95	17.18
**Father’s education**						
Primary school or less	58	21.09	66	23.74	124	22.42
Middle school/technical secondary school	186	67.64	177	63.67	363	65.64
Junior college/college and above	27	9.82	32	11.51	59	10.67
Don’t know	4	1.45	3	1.08	7	1.27
**Mother’s education**						
Primary school or less	84	30.55	91	32.73	175	31.65
Middle school/technical secondary school	167	60.73	168	60.43	335	60.58
Junior college/college and above	21	7.64	16	5.76	37	6.69
Don’t know	3	1.09	3	1.08	6	1.08
**Family income** a						
Below 999 RMB per month	54	19.85	58	20.86	112	20.25
1000 to 1999 RMB per month	93	34.19	108	38.85	201	36.35
2000 to 2999 RMB month	74	27.21	67	24.10	141	25.50
Above 3000 RMB month	51	18.75	42	15.11	93	16.82
Intention to smoke**						
Very unlikely	137	50.74	219	81.72	356	66.17
Unlikely	44	16.30	19	7.09	63	11.71
Likely	69	25.56	25	9.33	94	17.47
Very likely	20	7.41	5	1.87	25	4.65
**No. of Cigarettes smoked per day** a **						
0 cigarette	141	55.29	248	92.88	389	74.52
1 cigarette	34	13.33	12	4.49	46	8.81
2 to 5 cigarettes	63	24.71	6	2.25	69	13.22
More than 5 cigarettes	17	6.67	1	0.37	18	3.45

Note:

a Frequency not adding up to total due to missing data; gender difference,

** :*p* < 0.01(*χ*^2^ test)

**Table 2:** Item Response and Reliability of the PMT Scale for Tobacco Use among Chinese Adolescents (N=553).

Item and Primary Sub-constructs	N^a^	Mean (SD)	Correlation with total	Alpha/Alpha with deleted item
**Severity**	**551**	**5.88 (1.36)**		**0.76**
1. The earlier a person starts smoking, the greater the harm	549	6.25 (1.48)	0.54	0.72
2. More smokers get sickness than nonsmokers	551	5.74(1.77)	0.63	0.62
3. Smokers died earlier than nonsmokers	550	5.67 (1.69)	0.59	0.67
**Vulnerability**	**551**	**4.76 (1.37)**		**0.48**
4. I would become addict if I smoking	550	2.84 (2.11)	0.12	0.70
5. I would get sick if I smoke	550	5.41 (2.06)	0.44	0.12
6. If I smoke, I may die earlier	550	6.03 (1.67)	0.41	0.24
**Intrinsic Rewards**	**550**	**2.36 (1.44)**		**0.80**
10. Smoking makes people feel comfortable	550	2.49 (1.79)	0.58	0.79
11. Smoking helps people concentrate	549	2.46 (1.78)	0.70	0.66
12. Smoking enhances brainwork	549	2.12 (1.54)	0.66	0.72
**Extrinsic Rewards**	**551**	**2.71 (1.42)**		**0.61**
7. Smokers look cool and fashionable	551	2.48 (1.86)	0.41	0.51
8. Smoking is good for social networking	551	3.05 (2.05)	0.48	0.42
9. The life of a smoker is happier than a nonsmoker	550	2.59 (1.77)	0.37	0.58
**Self-Efficacy**	**550**	**5.94 (1.40)**		**0.73**
13. No one could persuade me if I do not want to smoke	549	5.59 (1.98)	0.53	0.68
14. Even if all who around me smoke, that do not mean I must smoke	548	6.13 (1.64)	0.59	0.59
15. I can refuse even if a relative or friend asks me to smoke	550	6.13 (1.54)	0.55	0.65
**Response Efficacy**	**550**	**5.14 (1.70)**		**0.68**
16. People will feel good by not smoking	549	4.68 (2.25)	0.45	0.65
17. People will be less likely to get disease if they do not smoke	550	5.37 (2.16)	0.58	0.47
18. Quit smoking is good for disease recovery	550	5.37 (2.09)	0.46	0.64
**Response Cost**	**550**	**2.58 (1.41)**		**0.59**
19. A person may be isolated if…does not smoke	550	2.02 (1.66)	0.43	0.45
20. Refusing a cigarette offer is very impolite	550	3.17 (2.06)	0.37	0.54
21. One will miss the enjoyment if he or she does not smoke	550	2.54 (1.97)	0.41	0.47

Note:

a Effective sample size; item-total correlation coefficients were all statistically significant (*p*<0.01).

**Table 3:** Correlation Coefficients assessing the association of pmt constructs with intention to smoking and the number of cigarettes smoked per day.

Construct	Boys (N=275)		Girls (N=278)		Overall (N=553)	
	*r*	*P*	*r*	*P*	*r*	*P*
**Intention to smoke**						
**Threat Appraisal**	0.316	<0.001	0.259	<0.001	0.353	<0.001
*Perceived Threat*	−0.197	0.001	−0.193	0.002	−0.226	<0.001
Vulnerability	−0.137	0.025	−0.162	0.008	−0.165	<0.001
Severity	−0.214	<0.001	−0.166	0.007	−0.231	<0.001
*Perceived Rewards*	0.270	<0.001	0.200	0.001	0.306	<0.001
Intrinsic rewards	0.302	<0.001	0.194	0.001	0.309	<0.001
External rewards	0.182	0.003	0.158	0.010	0.240	<0.001
**Coping Appraisal**	0.213	<0.001	0.116	0.059	0.251	<0.001
Self-efficacy	−0.171	0.005	−0.098	0.110	−0.192	<0.001
Response efficacy	−0.018	0.773	−0.063	0.306	−0.073	0.093
Response cost	0.223	<0.001	0.082	0.179	0.238	<0.001
**# of Cigarettes smoked**						
**Threat Appraisal**	0.430	<0.001	0.139	0.024	0.411	<0.001
*Perceived Threat*	−0.194	0.002	−0.016	0.790	−0.195	<0.001
Vulnerability	−0.114	0.070	−0.011	0.852	−0.117	0.008
Severity	−0.231	<0.001	−0.016	0.799	−0.223	<0.001
*Perceived Rewards*	0.446	<0.001	0.186	0.002	0.424	<0.001
Intrinsic rewards	0.419	<0.001	0.180	0.003	0.368	<0.001
External rewards	0.375	<0.001	0.150	0.014	0.387	<0.001
**Coping Appraisal**	0.306	<0.001	0.137	0.026	0.334	<0.001
Self-efficacy	−0.333	<0.001	−0.040	0.512	−0.292	<0.001
Response efficacy	−0.155	0.014	−0.146	0.017	−0.174	<0.001
Response cost	0.198	0.002	0.085	0.168	0.250	<0.001

**Note**: *r*’s are Pearson correlation coefficients.
